# Extracranial Facial Nerve Schwannoma Treated by Hypo-fractionated CyberKnife Radiosurgery

**DOI:** 10.7759/cureus.797

**Published:** 2016-09-21

**Authors:** Ayaka Sasaki, Shinichiro Miyazaki, Tomokatsu Hori

**Affiliations:** 1 Neurosurgery, Tokyo Women's Medical University; 2 CyberKnife Center, Shinyurigaoka General Hospital; 3 Department of Neurosurgery, Shinyurigaoka General Hospital

**Keywords:** neurofunctional preservation, cyberknife radiosurgery, extracranial facial nerve schwannoma

## Abstract

Facial nerve schwannoma is a rare intracranial tumor. Treatment for this benign tumor has been controversial. Here, we report a case of extracranial facial nerve schwannoma treated successfully by hypo-fractionated CyberKnife (Accuray, Sunnyvale, CA) radiosurgery and discuss the efficacy of this treatment.

A 34-year-old female noticed a swelling in her right mastoid process. The lesion enlarged over a seven-month period, and she experienced facial spasm on the right side. She was diagnosed with a facial schwannoma via a magnetic resonance imaging (MRI) scan of the head and neck and was told to wait until the facial nerve palsy subsides. She was referred to our hospital for radiation therapy. We planned a fractionated CyberKnife radiosurgery for three consecutive days. After CyberKnife radiosurgery, the mass in the right parotid gradually decreased in size, and the facial nerve palsy disappeared. At her eight-month follow-up, her facial spasm had completely disappeared. There has been no recurrence and the facial nerve function has been normal. We successfully demonstrated the efficacy of CyberKnife radiosurgery as an alternative treatment that also preserves neurofunction for facial nerve schwannomas.

## Introduction

Facial nerve schwannoma is a rare tumor composing less than two percent of intracranial benign tumors [1-2]. The treatment goal for this benign tumor is to prevent the tumor growth while preserving the function of the facial nerve. Complete surgical resection inevitably results in severe facial palsy in most cases, whereas radiosurgery for facial nerve schwannomas has been proven to be safe and successful in previous studies [1]. Hypo-fractionated radiosurgery is a method of staged irradiation with a decreased single dose and has successfully demonstrated efficacy in terms of preservation of both perifocal critical tissues and nerve function [3]. For the past four years, we have performed CyberKnife radiosurgery on some cranial nerve schwannomas, including vestibular schwannomas, followed by excellent outcomes. We present a case report of extracranial facial nerve schwannoma treated successfully by hypo-fractionated CyberKnife radiosurgery and discuss the efficacy of this treatment. Informed consent was obtained from the patient for this study.

## Case presentation

**Figure 1:**
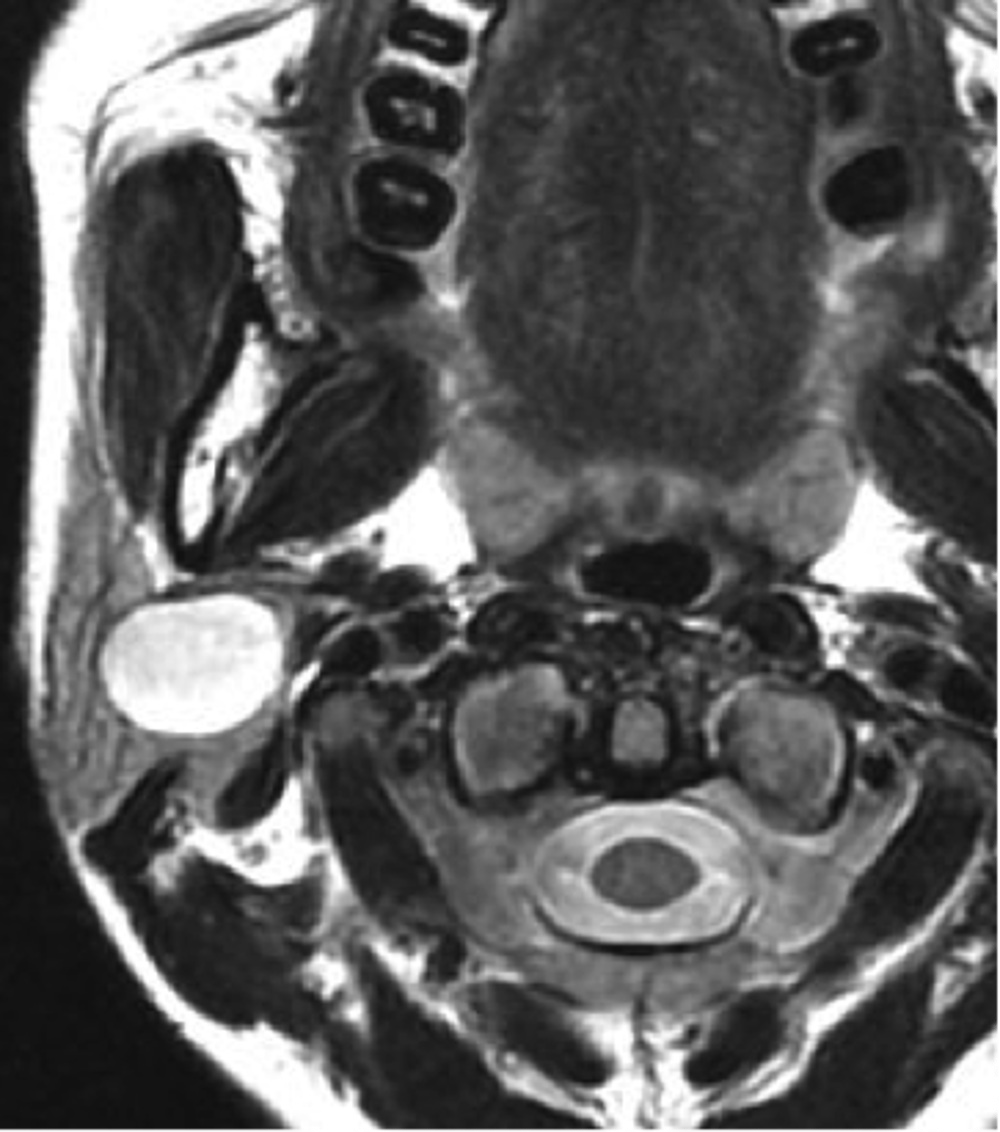
Axial T2-weighted image showing a heterogeneous, hyper-intense, cystic lesion in the right parotid.

A 34-year-old woman noticed a swelling in her right mastoid process approximately seven years before her first medical consultation regarding the condition. The lesion gradually enlarged and a facial spasm appeared on the right side of her face. She visited a city hospital and was diagnosed with a facial schwannoma via a brain and neck magnetic resonance imaging (MRI) scan (Figure 1).

She consulted multiple head-and-neck surgeons, and all of them told her to wait until the facial nerve palsy subsides. This is because surgical tumor resection inevitably causes facial nerve palsy and requires a scheduled neuroanastomosis. Two months later, she was referred to our hospital for radiation therapy. She had a facial spasm that was triggered by grimacing. She also exhibited reproducible right facial nerve palsy (House-Brackmann Grade I) induced by the compression of the mass in the right parotid. We planned a fractionated CyberKnife radiosurgery for three consecutive days aiming for good tumor control as well as preservation of the neural function.

**Figure 2:**
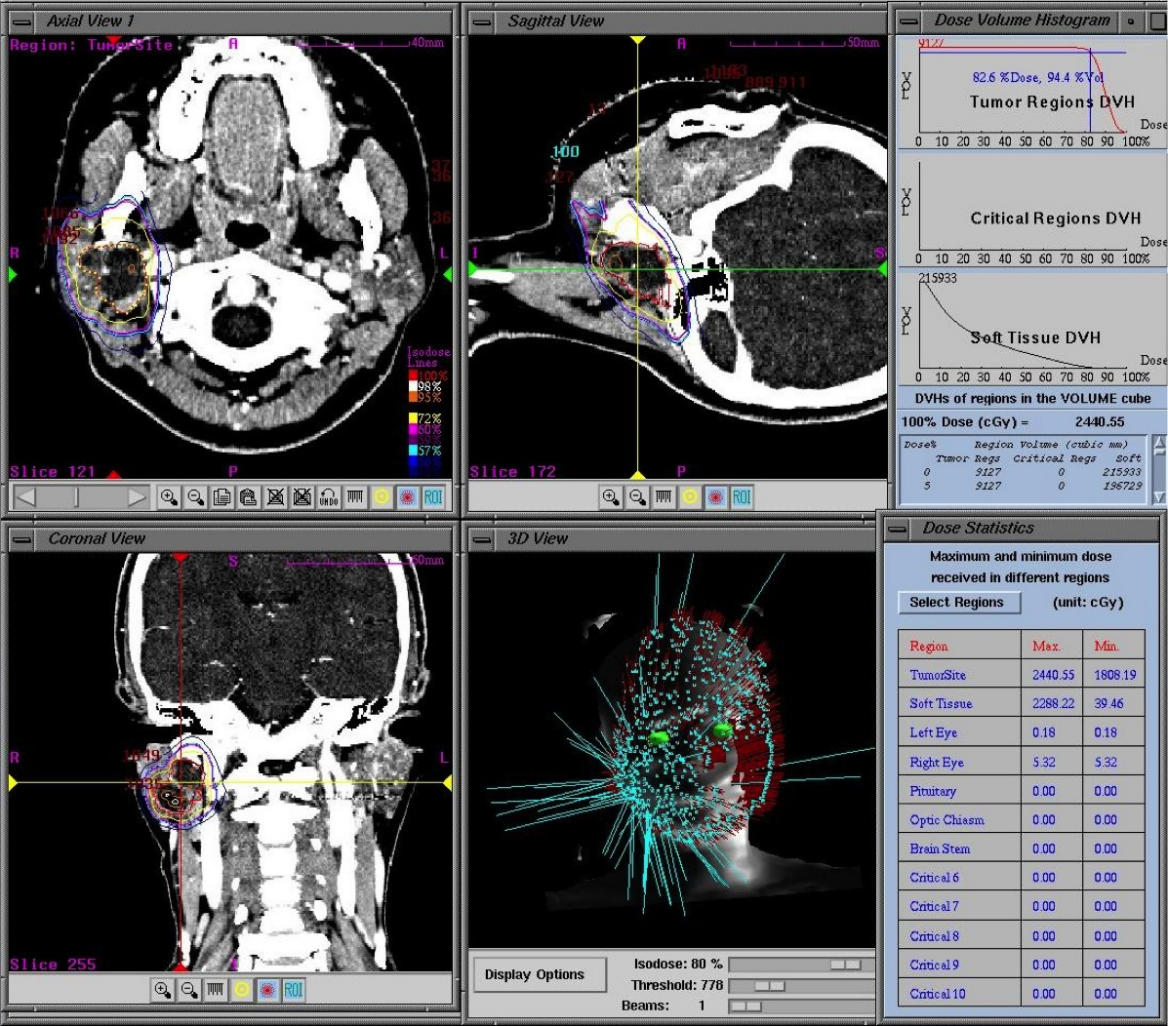
CyberKnife planning images obtained from contrast-enhanced CT images.

We planned the procedure based on computed tomography (CT) imaging and referenced an MRI as needed (Figure 2). The initial tumor volume was 9.1 mL. We prescribed 20.11 Gy of the marginal dose, which was divided into three equal doses of 6.7 Gy 82.4%D_95_ delivered in three consecutive stages at a 24-hour interval.

**Figure 3:**
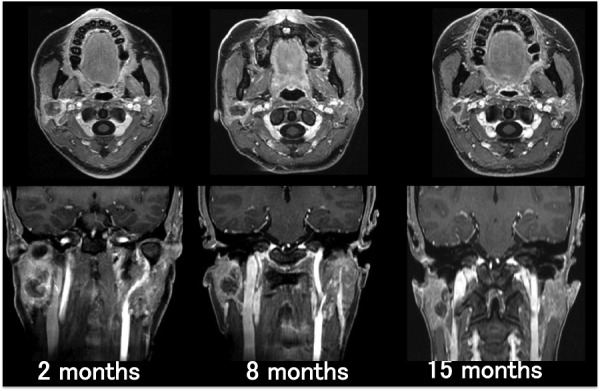
Time course of axial (upper row) and coronal (lower row) T1-weighted with Gd-enhancement. Over the 15-month follow-up period, the mass in the right parotid shows a decrease in size.

After CyberKnife radiosurgery, the mass in the right parotid gradually decreased in size from 9.1 mL to 2.1 mL over 15 months. The volume reduced to 23% of the original mass (Figure 3). The facial nerve palsy disappeared and was not induced by compression of the mass. 

At her eight-month follow-up, her facial spasm had completely disappeared. There was no recurrence and the facial nerve function has been normal. The treatment did not require hospital admission.

## Discussion

Jannetta reported that a cause of hemifacial spasm was neurovascular compression [4], that is, a compression of the intracranial facial nerve by a vessel or a tumor at its root exit zone [5-6]. Typical facial spasm due to an extracranial tumor is extremely rare [7-8], and this report represents the first reported case of extracranial facial nerve schwannoma in the literature, to our knowledge.

There are novel treatments for facial schwannoma such as microsurgery (via the translabyrinthine or transmastoid approach) and radiosurgery [9-10], however, “wait and scan” observation is also an option. Even with highly advanced surgical techniques, the complete surgical resection of the tumor is inevitably followed by facial nerve palsy. Interposition of the nerve graft or hypoglossofacial anastomosis is usually performed, but facial nerve palsy remains no better than House-Brackmann Grade III [2]. The strength of the facial nerve is 80% regained, on average [2]. In addition, subsequent complications such as abnormal synkinesis may occur even after a recovery from facial nerve palsy. Given such a high operative risk for severe facial nerve palsy and potential deterioration in the quality of life for the patient, a “wait and scan” approach is not recommended until the facial nerve palsy subsides.

When treating benign tumors such as facial schwannomas, it is crucial to take into consideration not only the tumor removal and its control, but also the preservation and recovery of facial nerve function and treatment-induced complications. In this case, we opted for a fractionated CyberKnife radiosurgery strategy, using which we had achieved good tumor control as well as preservation of the neural function in our previous series of intracranial schwannomas such as vestibular schwannomas, trigeminal schwannomas and facial schwannomas. Multisession CyberKnife radio surgery allows us to treat schwannomas with multiple shots of a dose, which does not cause adverse effects. 

In previous studies, stereotactic radiosurgery has been successful in controlling tumor growth and preserving neural function in intracranial facial nerve schwannoma and vestibular nerve schwannoma [1]. Hypo-fractionated CyberKnife radiosurgery should be effective for large tumors or extracranial tumors. This treatment is recommended for the early clinical stage of the tumor. This also means that there is no limitation of indicative stage for the use of CyberKnife radiosurgery. More case studies with longer follow-up are necessary to establish the appropriate treatment with CyberKnife.

## Conclusions

We successfully treated the extracranial facial nerve schwannoma by hypo-fractionated CyberKnife radiosurgery resulting in a dramatic reduction of the tumor in eight months. The patient’s hemifacial paresis and hemifacial spasm disappeared and the facial nerve function became normal. Hypo-fractionated CyberKnife radiosurgery could benefit patients with enlarging tumors or tumors that become symptomatic with worsening facial nerve function. In terms of tumor control and preservation of neural function with minimal complications, CyberKnife radiosurgery is a favorable alternative treatment for extracranial facial nerve schwannoma together with other radiosurgical therapies.
